# Intracellular processing of silica-coated superparamagnetic iron nanoparticles in human mesenchymal stem cells†
†Electronic supplementary information (ESI) available. See DOI: 10.1039/c8ra09089k


**DOI:** 10.1039/c8ra09089k

**Published:** 2019-01-23

**Authors:** Richard P. Harrison, Veeren M. Chauhan, David Onion, Jonathan W. Aylott, Virginie Sottile

**Affiliations:** a Wolfson Centre for Stem Cells, Tissue Engineering and Modelling (STEM), School of Medicine, Nottingham, NG7 2RD, UK. Email: Virginie.Sottile@nottingham.ac.uk; b Centre for Biological Engineering, Loughborough University, Leicestershire LE11 3TU, UK; c School of Pharmacy, University of Nottingham, Boots Sciences Building, University Park, Nottingham, NG7 2RD, UK. Email: Jon.Aylott@nottingham.ac.uk; Email: veeren.chauhan@nottingham.ac.uk; d University of Nottingham Flow Cytometry Facility, School of Life Sciences, University of Nottingham, NG7 2UH, UK

## Abstract

Silica-coated superparamagnetic iron nanoparticles (SiMAGs) are an exciting biomedical technology capable of targeted delivery of cell-based therapeutics and disease diagnosis. However, in order to realise their full clinical potential, their intracellular fate must be determined. The analytical techniques of super-resolution fluorescence microscopy, particle counting flow cytometry and pH-sensitive nanosensors were applied to elucidate mechanisms of intracellular SiMAG processing in human mesenchymal stem cell (hMSCs). Super-resolution microscopy showed SiMAG fluorescently-tagged nanoparticles are endocytosed and co-localised within lysosomes. When exposed to simulated lysosomal conditions SiMAGs were solubilised and exhibited diminishing fluorescence emission over 7 days. The *in vitro* intracellular metabolism of SiMAGs was monitored in hMSCs using flow cytometry and co-localised pH-sensitive nanosensors. A decrease in SiMAG fluorescence emission, which corresponded to a decrease in lysosomal pH was observed, mirroring *ex vivo* observations, suggesting SiMAG lysosomal exposure degrades fluorescent silica-coatings and iron cores. These findings indicate although there is a significant decrease in intracellular SiMAG loading, sufficient particles remain internalised (>50%) to render SiMAG treated cells amenable to long-term magnetic cell manipulation. Our analytical approach provides important insights into the understanding of the intracellular fate of SiMAG processing, which could be readily applied to other particle therapeutics, to advance their clinical translation.

## Abstract 2

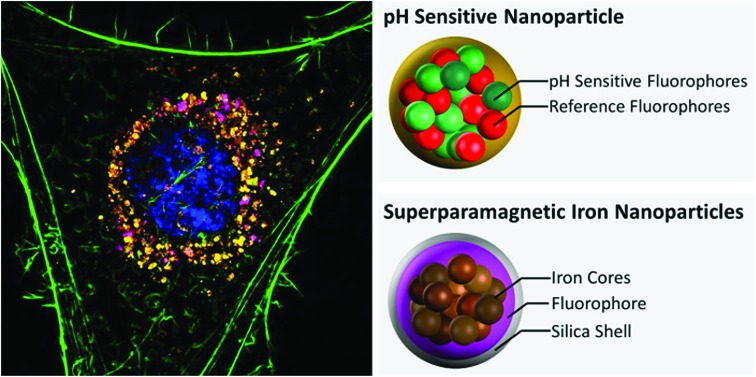
Elucidating the intracellular fate of silica-coated superparamagnetic iron nanoparticles (SiMAGs) using fluorescent pH-sensitive nanosensors, super-resolution fluorescence microscopy and particle counting flow cytometry.

## Introduction

The biomedical uses of magnetic particles have evolved to include a variety of applications. Many of these novel practices deliver an active therapeutic benefit, whilst providing diagnostic information.^1^

Superparamagnetic nanoparticles are a class of iron nanoparticle, which are typically formed from magnetite (Fe_3_O_4_) and/or maghemite (γ-Fe_2_O_3_). One of the major advantages of superparamagnetic nanomaterials is the complete absence of magnetism following the removal of external magnetic fields to produce a material that avoids agglomeration and remains colloidally stable.^2^ In addition, due to their flexibility in design and targeted interaction with biological systems the particles have also demonstrated therapeutic and diagnostic potential in experimental *in vivo* studies.^3^ These properties open the possibility for targeted delivery of cells loaded with superparamagnetic nanoparticles, through utility of magnets at the required site of action,^4^ as well as excellent MRI contrast agents to facilitate prognosis and diagnosis of disease.^5^ For example, human mesenchymal stem cells (hMSCs) are one of the most promising cell types for regenerative medicine for the treatment of diseases,^6^,^7^ such as rheumatoid and osteoarthritis.^8^ hMSCs readily uptake silica iron paramagnetic (SiMAG) particles, such that loaded cells have the dual advantage in tissue engineering and regenerative medicine (TERM) therapy of targeted delivery *via* application of an external magnetic field, whilst being readily trackable by MRI.^3^,^9^ However, despite SiMAGs promise for *in vivo* applications a comprehensive understanding of their long-term cellular fate and the consequential health implications are yet to be determined.

It has been suggested that predictive models capable of determining particle toxicity require a systematic understanding of the fate, kinetics, clearance, metabolism, protein coating, immune response and toxicity parameters.^10^ To facilitate modelling of particle toxicity the measurement of internal cellular kinetics of key molecules and ions such as pH,^11^ glucose,^12^ lactate^13^ and oxygen,^14^ which have profound effects on cell response to particle loading, could progress our knowledge of cell systems and the potential of TERM therapy.

Current assessment systems are often limited by throughput of the products and many are destructive or sample altering in nature.^15^ Previous micro-sensory approaches focussed on miniaturising existing sensory elements, such as microelectrodes^16^ or fibre optic sensors,^17^ which can cause substantial damage to biological systems. Therefore, a range of smarter measurement systems for TERM have been produced.^18^–^20^

**Fig. 1:**
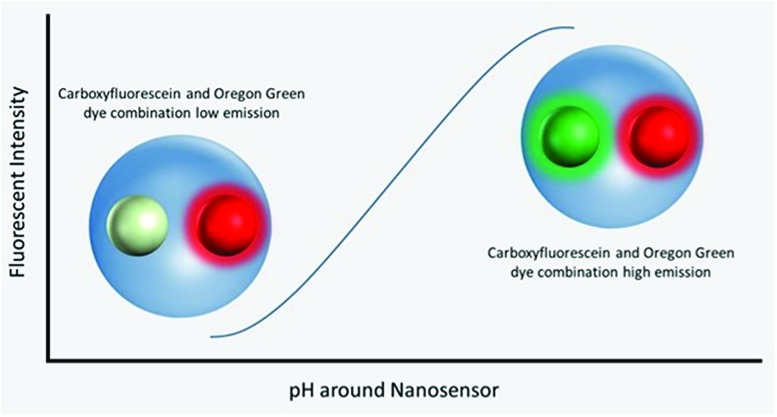
Visualisation of nanosensors mechanism of action. Dynamic extended range nanosensors have a linear range in physiological pH ranges in which the emission of the carboxyflourescein and Oregon Green® dye combination increases as pH increases. The red dye meanwhile is unaffected and acts as a reference point for normalising fluorescence emission.

Polyacrylamide-based fluorescent nanosensors are an example of a smart measurement system that allow for complex sensory data to be acquired with minimal sample interference.^21^ They are spherical particles, ∼50 nm in diameter, which allow for many particles to be delivered to intracellular spaces and provide a high signal-to-noise ratio.^22^ Due to their size and inert matrix, combined with their ratiometric measurement properties, they can collect valuable subcellular real-time metrics for parameters, such as pH and oxygen (Fig. 1).^23^,^24^

Fluorescent extended dynamic range pH-sensitive nanosensors are inert spherical probes prepared from polyacrylamide, that have a particle diameter centred at ∼50 nm (Fig. 1). They are covalently linked to two fluorescein-based pH-sensitive fluorophores (*λ*_em_ max 520 nm) and a rhodamine-based pH-sensitive reference fluorophore (*λ*_em_ max 577 nm).^25^ The fluorescein-based fluorophores carboxyflourescein and Oregon Green® are combined in a 1 : 1 ratio and exhibit a diminishing fluorescence emission from pH 7.5 to 3.5.^24^ When these fluoresceins are combined with the pH-insensitive reference 5-(and-6)-carboxytetramethylrhodamine to produce fluorescent extended dynamic range pH-sensitive nanosensors, they can be used to make accurate ratiometric measurements that are independent of fluorophore concentration, fluctuations in excitation energy, as well as detector sensitivity and light scattering.^26^ For example, extended dynamic range pH-sensitive nanosensors have been shown to be capable of making accurate ratiometric measurements to a high spatial (<50 nm), temporal (<100 ms) and pH resolution (±0.17 pH units) in the model organisms *Caenorhabditis elegans*,^27^ *Pristionchus pacificus*^28^ and *Saccharomyces cerevisiae*.^21^

This article investigates the utility and safety of SiMAGs for *in vivo* applications, by determining their degradation profile in simulated lysosomal conditions as well as their *in vitro* intracellular fate in hMSCs using extended dynamic range pH-sensitive fluorescent nanosensors. SiMAG particles were characterised using dynamic light scattering (DLS), scanning electron microscopy (SEM), transmission electron microscopy (TEM), and flow cytometry. The uptake profile, toxicity and cellular fate were determined by particle counting flow cytometry and super resolution fluorescence microscopy. Finally, particle degradation was monitored in simulated lysosomal conditions, as well as in human mesenchymal stem cells (hMSCs) and macrophages alongside extended dynamic range pH-sensitive nanosensors, to determine particle degradation kinetics.

## Results

SiMAGs are heterogenous particles and can be obtained in a variety of sizes, morphologies and fluorophores. This heterogeneity can influence how they interact with biological systems. Therefore, prior to determining mechanisms of intracellular processing, SiMAGs were characterised for their physical, fluorescence and biocompatible properties.

**Fig. 2:**
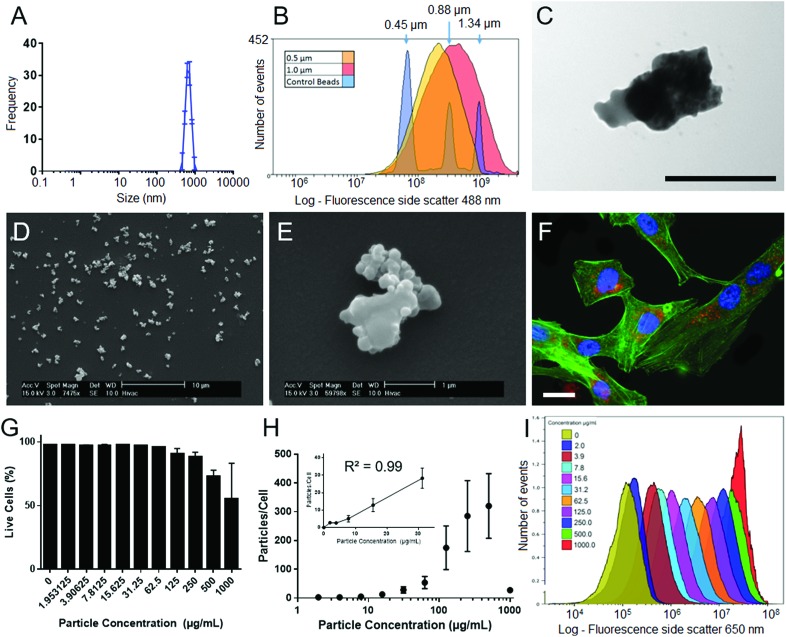
Particle characterisation and cell loading characteristics of SiMAG particles. (A) Dynamic light scattering (DLS) sizing data of SiMAG reveals a tight clustering around the 1000 nm range. (B) Flow cytometry sizing was utilised to confirm this finding and show a similar population spread as assessed by side scatter. (C) TEM imaging demonstrates the internal characteristics of the SiMAG particles highlighting clearly the internal iron cores (black scale bar 500 nm). (D) Low and (E) high magnification images of SiMAG particles. (F) SiMAG particles can be loaded into MSCs and observed within the intracellular space as shown in the fluorescent image of cells loaded with SiMAG (red) counter stained with phallodin (cytoskeleton, green) and Hoechst 33342 (nucleus, blue), where scale bar = 20 μm. (G) Cell viability of hMSCs loaded with increasing doses of SiMAG particles determined by membrane permeability assay using 7-AAD and flow cytometric analysis. (H) The dose–response of particle loading in to hMSCs by flow cytometry based on median Nile-Red fluorescence of cells and particles. (I) Labelling efficiency of cells loaded with increasing concentrations SiMAG assessed using flow cytometry.

The physical properties of SiMAG particles were characterised with DLS, flow cytometry, SEM and TEM. DLS showed SiMAG size is centred at 712 nm with sizes ranging between 450–1000 nm (Fig. 2A). This was confirmed with flow cytometry side scatter analysis which demonstrated sizes were centred around 1000 nm (Fig. 2B). TEM confirmed the SiMAGs are composed of a silica coating that envelopes dense iron cluster cores (Fig. 2C). SEM highlighted SiMAGs are clustered together into large structurally diverse particles (∼1000 nm, Fig. 2D and E) that consist of 50–100 nm iron nanostructures. The fluorescence properties of SiMAGs was characterised using spectrophotometry which showed their fluorescence excitation maxima (*λ*_ex_ max) and emission maxima (*λ*_em_ max) are 633 nm and 672 nm, respectively.

To investigate SiMAG cytocompatibility and determine optimal working, hMSCs were labelled with SiMAG particles (Fig. 2F) up to a concentrations of 1000 μg mL^–1^ (w/v). Viability was determined using the live/dead fluorescent dye 7-AAD (Fig. 2G). hMSCs showed excellent tolerance of the loading process with only the high SiMAG particle concentrations (>100 μg mL^–1^) reducing cell viability (Fig. 2G). Similarly, cell morphology was unaffected with doses up to 100 μg mL^–1^ (Fig. S1†). SiMAG particle uptake was quantified using flow cytometric analysis of both free particles and loaded cells, which was used to calculate the number of delivered particles per cell (Fig. 2H) as well as time-lapse identification of uptake (ESI Movie†). Linear dose dependent loading for SiMAG particles was observed up to 30 μg mL^–1^ (Fig. 2H inset). A dose of 10 μg mL^–1^, corresponding to 8 ± 4 particles per cell, was selected for subsequent experiments due its low toxicity, which was ∼10 fold lower than any observable loss in cellular viability (Fig. 2I).

Furthermore, previous studies have demonstrated a concentration of 10 μg mL^–1^ also provides optimal magnetic spatial control and MRI tracking.^3^

**Fig. 3:**
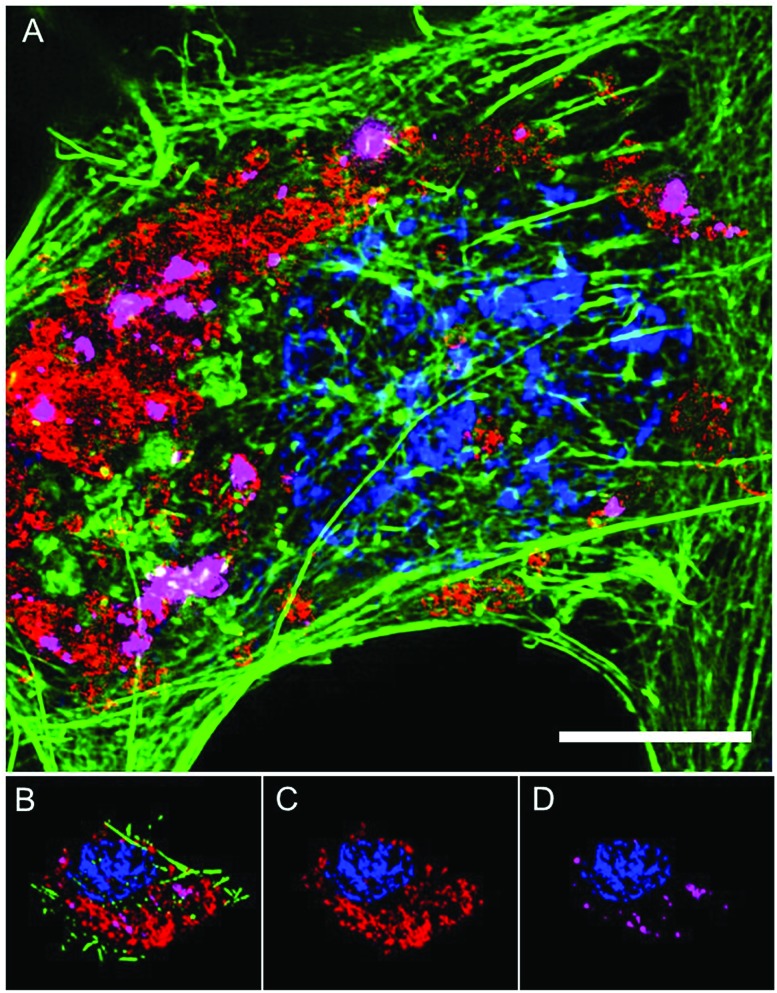
Super resolution microscopy showing localisation of SiMAG particles and lysosomes. hMSCs pre-transfected with LAMP-1-RFP fusion (red) and loaded with SiMAG particles (pink) were stained with phalloidin (green) and Hoechst 33342 (blue) to identify actin cytoskeleton and nucleus respectively. (A) Accumulation of SiMAG in LAMP-1 positive peri-nuclear cytoplasmic space. (B–D) Particle distribution by 3D reconstruction (B) cytoskeleton, nucleus, LAMP-1 and SiMAG. (C) Nucleus and LAMP-1. (D) Nucleus and SiMAG. Scale bar (A) = 20 μm.

The intracellular location of SiMAG particles following uptake into hMSCs was determined using super resolution structured illumination microscopy (SIM). SiMAGs were found to reside in sub-cellular compartments adjacent to the nucleus, consistent with the location of lysosomes (Fig. 3). Lysosomes provide a degrading environment for foreign matter through the maintenance of an acidic pH,^29^ which provides optimal conditions for hydrolytic enzymes.^30^ Therefore, to confirm the co-localisation of SiMAGs with lysosomes, hMSCs loaded with particles were counterstained with lysosomal associated membrane protein 1 (Lamp1) and imaged using SIM. Three dimensionally reconstructed images showed that internalised SiMAG particles were co-localised with lysosomes in the intracellular space surrounding the nucleus (Fig. 3B–D and ESI Fig. S2 and S3†).

**Fig. 4:**
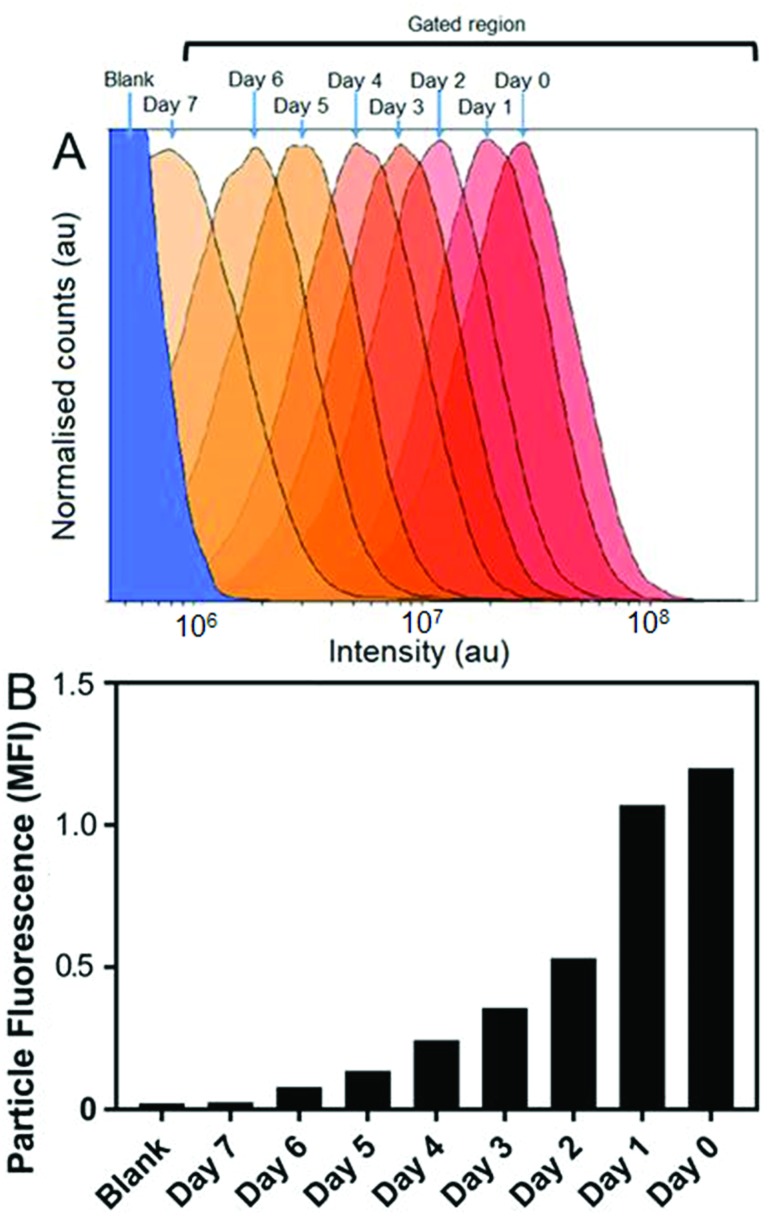
Simulated lysosomal conditions lead to particle degradation over time. SiMAG particles were incubated in low pH buffer (pH 4.5) and the fluorescent intensity of >10 000 particles assessed daily by flow cytometry. (A) Histogram overlay plots showing intensity distribution over time and (B) corresponding mean fluorescence intensities.

Cellular micro-environments, such as lysosomes, are challenging to characterise due to their inherent biological complexity. Therefore, biologically simulated environments can be used excellent model to guide *in vitro* or *in vivo* studies.^31^ Bearing this in mind, the effects of simulated lysosomal conditions (acidic sodium acetate and hydrochloric acid buffer at pH 4.5)^32^ on SiMAG particle processing was studied using flow cytometry and Inductively Coupled Plasma Mass Spectrometry (ICP-MS). Under simulated lysosomal conditions SiMAG particles demonstrated a progressive loss of fluorescence signal over 7 days (Fig. 4A and B). Whereas, ICP-MS exhibited a subtle reduction in the amount of iron that was released from degrading SiMAG particles (Fig. S4†). These observations suggest the acidic conditions disrupt the silica-based surface chemistry of SiMAGs but have limited effect on the iron core. While these conditions simulate lysosomal pH, it is important to note this approach does not account for the degradative properties of the host of diverse of hydrolytic enzymes present within lysosomal vesicles.^33^

**Fig. 5:**
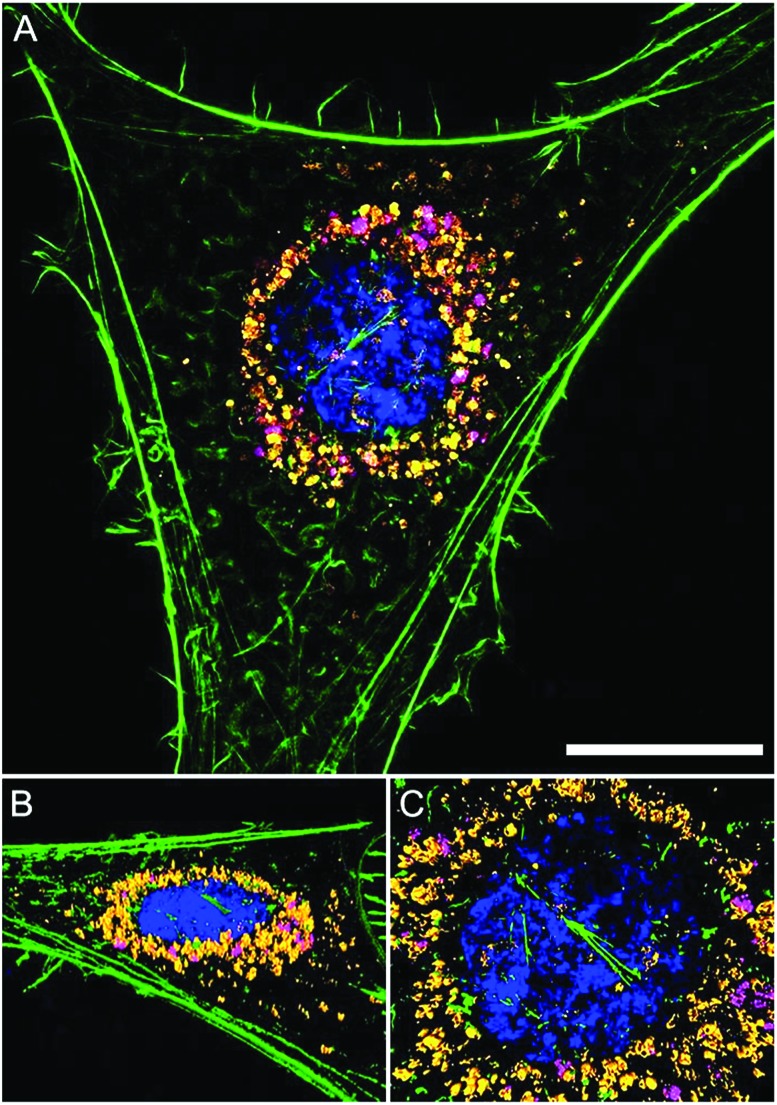
Super resolution microscopy demonstrates co-localisation of pH nanosensors and SiMAG particles in hMSCs. hMSCs loaded with pH nanosensors (gold) and SiMAG particles (pink) were stained with phalloidin (green) and Hoechst 33 342 (blue) to identify actin cytoskeleton and nucleus respectively. (A) Peri-nuclear accumulation of pH nanosensors and SiMAG particles. (B) 3D reconstruction demonstrates both particles to be in the same plane. (C) Particles tightly co-located around the nucleus. Scale bar = 10 μm.

To translate the simulated lysozyme findings into a cellular context, *in vitro* studies using hMSCs were conducted. Monitoring the lysosomal pH during SiMAG processing was facilitated using the smart measurement system of fluorescent extended dynamic range pH sensitive nanosensors. Nanosensors were examined for their size, cytotoxicity and loading in hMSCs (Fig. S5†). Nanosensors were shown to be well tolerated at high concentrations (>1000 μg mL^–1^). Super resolution microscopy was used to examine the distribution of both nanosensors and SiMAG particles within the cell (Fig. 5A). SiMAGs and pH-sensitive nanosensors, were found to be distributed around the nucleus, on the same plane within the centre of the cell (Fig. 5B and C). The co-localisation of both particles permitted the assessment SiMAGs processing using pH-sensitive nanosensors and flow cytometry in the lysosomal pH microenvironment.

**Fig. 6:**
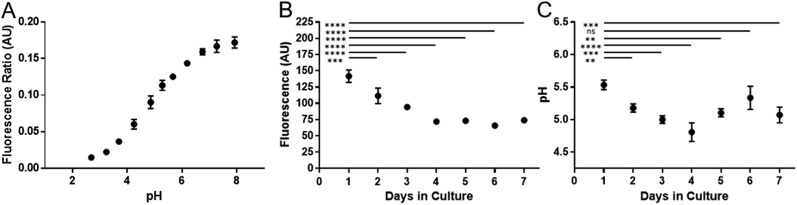
Temporal signal loss of SiMAG particles and acidification as measured by pH nanosensors in dual loaded hMSCs over seven days. (A) *In situ* pH calibration curve generated from hMSCs re-suspended in pH buffers in the presence of the ionophore nigericin. (B) Loss of Nile-Red fluorescence over time in SiMAG/pH nanosensors loaded hMSCs (C) pH measurements in hMSCs dual loaded with SiMAG/pH nanosensors.

Cell division has been shown to influence nanoparticle labelling approaches^34^ and could dilute the concentration of nanoparticles in the cell.^35^ To minimise this effect, hMSCs were grown to confluence prior to labelling to ensure further cell division was minimised. Growing cells to confluency was chosen in preference to Mitomycin C cell cycle inhibition, as the latter demonstrably altered the phenotype of the cells (data not shown). hMSCs were initially labelled with SiMAGs (10 μg mL^–1^) and fluorescent extended dynamic range pH-sensitive nanosensors (100 μg mL^–1^) for 24 hours. Hereafter, the hMSCs were washed, to remove excess SiMAGs and nanosensors which were monitored for 7 days to determine the particle processing. An *in situ* pH calibration was conducted by suspending hMSCs in potassium (K^+^) rich buffer solutions, ranging from pH 8.0 to 3.5, and incubation with nigericin, which catalyses the electroneutral exchange of K^+^ and hydrogen (H^+^) (Fig. 6A).^36^

The *in vitro* studies showed there was an accelerated loss of SiMAG fluorescence emission from day 1 to 4 (Fig. 6B), which was subsequently followed by emission stabilisation from day 4 to 7. This *in vitro* decrease in SiMAG fluorescence emission *in vitro* mirrors the observations from simulated lysosomal suspensions (Fig. 4). The time dependent decrease in fluorescence signal of the SiMAGs also coincided with a decrease in its pH microenvironment from day 1 to 4, from pH 5.43 ± 0.06 to 4.81 ± 0.14, which was followed by an increase in pH on day 6 to 5.33 ± 0.17 (Fig. 6C).

SiMAG particles demonstrate a non-significant increase in fluorescence in low pH environments (Fig. S6†), such that particles traversing the acidic endo-lysosomal pathway would exhibit an apparent enhanced fluorescence emission. Therefore, we postulate that the SiMAG particles are progressively processed, *via* exposure to the low pH microenvironment experienced in the lysosome from day 1 to 4. After particle processing the lysosomal pH recovers to pre SiMAG dosed levels, as indicated by the pH-sensitive nanosensors. Our hypothesis is supported by studies that have demonstrated lysosomes gradually acidify and process foreign matter, although there is a degree of heterogeneity and lack of synchronicity in this process.^32^,^37^,^38^ Similarly, the heterogeneity in particle number within each lysosome may affect the dissolution rate for specific sub-populations of particle-lysosome groups. These results suggest SiMAG lysosomal exposure degrades both fluorescent silica-coatings and iron cores. Based on our particle loading calibration graph, these observations also indicate the average particle loading has reduced by no more than 50% (∼5 particles) over 7 days. Critically, this small reduction in particle number would still leave the cells well within the useable range for magnetic cell manipulation.^3^

## Conclusion

In summary, through application of super resolution structured illumination microscopy, the subcellular location of the SiMAG particles was determined in unprecedented detail, in relation to the cellular nucleus, lysozymes, cytoskeleton and co-administered pH sensitive nanosensors. These findings showed SiMAGs are co-localised with both lysosomes and pH sensitive nanosensors around the nucleus. The analysis methodology developed in this article allows examination of the degradation profile for SiMAG particles endocytosed by hMSCs (×10^6^ cells) utilising the rapid fluorescent analysis afforded by multi-colour flow cytometry and the high sensitivity of extended dynamic range pH sensitive nanosensors. This powerful method can provide real time pH quantification for individual cells within a large population. The intracellular pH was mapped in cell populations over 7 days, which were correlated with a reduction in particle fluorescence emission, thus providing key insights into the intracellular processing of SiMAG. Moreover, our findings suggested over 7 days enough SiMAG remain in sub-cellular spaces such that their continual therapeutic and diagnostic potential can be exerted. Complementary analyses could also be applied to particles, with a variety of particle sizes and chemistry, to determine how cellular and lysosomal processing may differentially induce chemical changes on theranostic systems. Taken together, these results demonstrate the development of new measurement modalities, applicable to TERM products, that enhance our understanding of the complex interactions between theranostics particles and cellular processes.^39^ We envisage these methods could be adapted by nano, analytical and biological communities to better understand the clinical benefit of particle-based therapeutics and diagnostics at the cellular level.

## Experimental

The datasets generated during and/or analysed during the current study are available from the corresponding author on reasonable request. The human mesenchymal stem cells (hMSCs) used in this study are an immortalised clonal mesenchymal cell line of bone marrow origin,^40^ that have been used in a variety of experimental studies.^3^,^41^,^42^ All human cellular material was obtained in accordance with relevant guidelines and appropriate consent. All methods were carried out with relevant guidelines and regulations.

### Cell culture

Human bone marrow derived mesenchymal stem cell line (hMSCs)^41^,^42^ were cultured under standard conditions (37.5 °C, 5% CO_2_) in DMEM supplemented with 10% (v/v) FBS, 1% (v/v) non-essential amino acids, 1 mM l-glutamine, 1 mM pyruvate and 1% penicillin/streptomycin. Media was exchanged every other day for maintenance and post loading of cell monolayers. Cells were passaged using trypsin/EDTA.

### Scanning electron microscopy

Particle suspensions were deposited on 13 mm diameter Thermanox™ (Thermo Fisher Scientific, USA) coverslips and left to dry. Dehydrated coverslips were carbon coated with an Edwards 306 Vacuum Coater (Edwards, UK). Scanning was performed using a Philips XL30 FEG ESEM (FEI Company, USA) at an accelerating voltage of 5–15 kV.

### Brightfield microscopy

Cells labelled with SiMAG and pH nanosensors at increasing concentrations were observed for morphological changes and signs of stress. Brightfield images were acquired using an Eclipse TS100 inverted microscope (Nikon, Japan).

### Immunofluorescence & super-resolution imaging

hMSCs were cultured in 35 mm cat 1.5 glass bottom culture dishes (MatTek Corporation, USA). For lysosomal identification cell were exposed to CellLight® Lysosome RFP Bacmam 2.0 for 8 hours and left for a further 24 hours before immunostaining. Cells were fixed in 4% (v/v) PFA (VWR, UK), (15 min, room temperature) washed and permeabilised with 0.1% Triton x-100 for 5 min followed by two phosphate buffered saline (PBS) washes. Cells were labelled with Alexa Fluor® 488 phalloidin (6.6 μM in methanol) 15 minutes, room temperature and washed twice with PBS. Hoechst 33342 counterstain was added at 1 mM concentration and incubated for 15 minutes before washing. Cells were imaged by structured illumination (SIM) with an Elyra PS1 super resolution microscope (Zeiss, Switzerland) as a stack, which permits visualisation of structures beyond the resolution of conventional light microscopy.^43^

### Particle degradation analysis

Previous studies have investigated the influence of protein and biomolecular coronas on particle surfaces, acting as cellular protectants prior to their intracellular processing.^44^,^45^ This article examines the whole particle kinetics in serum-free conditions to minimise the confounding effects of protein corona. Fluorescently tagged SiMAG MP 1 μm, (screenMAG, colour R) (Chemicell GmbH, Germany) were added to a 50 : 50 mix of 1 M sodium acetate and 1 M hydrochloric acid to simulate the interior of a lysosome at pH 4.5. Suspensions of particles were removed each day for 7 days and each time washed three times with PBS to neutralise acidic conditions and remove digested constituents before flow cytometric analysis and ICP-MS.

### Labelling assessment of pH and SiMAG

hMSC cells were grown to confluency to ensure even uptake per cell density. SiMAG only, pH nanosensor only and SiMAG/pH nanosensors combined were added to monolayers at varying concentrations (0, 1.95, 3.91, 15.63, 31.25, 62.5, 125, 250, 500, 1000 and 2000 μg mL^–1^ of iron for SiMAG and total weight for pH nanosensors respectively). Uptake was performed over 24 hours in serum free conditions as it was previously found this greatly improves uptake.^3^ Excess particles were removed by washing three times with PBS and harvesting with trypsin/EDTA. To assess viability by membrane integrity assay cell suspensions were incubated with 5 μg mL^–1^ 7-AAD for 30 min at room temperature and analysed on an Astrios EQ Cytometer (Beckman Coulter, USA).

### pH sensitive nanoparticle measurement

Standards for internal cell pH calibration curve were generated by immersing cells in pH buffer solutions with the ionophore nigericin to equilibrate intracellular pH with extracellular pH.^7^ Cells were assessed by flow cytometry, with ratiometric measurements in test samples converted to pH units by regression analysis from the standard pH curve.

### Flow cytometry

SiMAG and pH nanosensors were added to PBS (30 μg mL^–1^) and suspended with vortexing. Using an Astrios EQ flow cytometer (Beckman Coulter) SiMAG particles were identified by 488 nm forward (mask M2) and side scatter analysis and Nile blue fluorescence was measured using 642 nm laser excitation and collection through a 671/30 band pass filter. pH nanosensors particles were identified by TAMRA fluorescence (reference dye) as excited by 562 nm laser and collected through a 614/20 nm band pass filter. pH sensitive FAM/Oregon Green fluorescence was measured following excitation with 488 nm and detection through a 513/26 nm band pass filter. Single colour polyacrylamide nanoparticles and Nile blue MP or cells loaded with these particles were used to determine compensation as (Fig. S5†) appropriate. Matched unlabelled and labelled MP and polyacrylamide nanoparticles were used to determine baseline median per particle fluorescence intensity (Nile blue and TAMRA respectively). Data was analysed using Summit (version 6.2) and Kaluza (version 1.5) software (Beckman Coulter, USA).

## Associated content

Please see ESI† for results showing (1) optical images for SiMAG and pH sensitive nanosensor loading in hMSCs, (2) co-localisation analysis for SiMAG and Lamp1, (3) pH-sensitive nanosensor characterisation, cellular viability and particle counting flow cytometric analysis (4) fluorescence response of SiMAG particles (Nile blue) to changes in pH and (5) ICPMS simulated lysosomal SiMAG degradation data.

## Author contributions

RPH, VMC & DO prepared manuscript, ESI† and assembled figures. RPH prepared the hMSC, macrophages and characterised SiMAGs particles (SEM & cell viability). VMC synthesised and characterised extended dynamic range pH sensitive fluorescent nanosensors (spectrophotometry & SEM). RPH and VMC conducted super resolution microscopy with technical support. RPH, VMC and DO conducted flow cytometry experiments. RPH, VMC, DO, JWA and VS contributed together towards scientific planning, direction, discussion and manuscript finalisation.

## Conflicts of interest

The authors declare no conflicts of interests.

## Supplementary Material

Supplementary information

Supplementary movie
