# Supplementary material for: Intracellular processing of silica-coated superparamagnetic iron nanoparticles in human mesenchymal stem cells
Source: RSC Adv. 2019 Jan 23;9(6):3176–84. doi: 10.1039/c8ra09089k (PMC6350623; doi:10.1039/c8ra09089k)
Supplement: RA-009-C8RA09089K-s001 [file RA-009-C8RA09089K-s001.pdf]

Electronic Supplementary Material (ESI) for RSC Advances.
This journal is © The Royal Society of Chemistry 2019

SUPPORTING INFORMATION

INTRACELLULAR PROCESSING OF SILICA-COATED SUPERPARAMAGNETIC IRON NANOPARTICLES IN HUMAN MESENCHYMAL STEM CELLS

Richard P Harrison [a, b†*], Veeren M Chauhan [c†*], David Onion [d]
Jonathan W Aylott [c*] & Virginie Sottile [a*]

[a] *Wolfson Centre for Stem Cells, Tissue Engineering and Modelling (STEM), School of Medicine, Nottingham, NG7 2RD, UK*
[b] *Centre for Biological Engineering, Loughborough University, Leicestershire, LE11 3TU, UK*
[c] *School of Pharmacy, University of Nottingham, Boots Sciences Building, University Park, Nottingham, NG7 2RD, UK*
[d] *University of Nottingham Flow Cytometry Facility, School of Life Sciences, University of Nottingham, NG7 2UH, UK*

†Authors contributed equally to work, *Corresponding Authors
Veeren.Chauhan@nottingham.ac.uk, Jon.Aylott@nottingham.ac.uk & Virginie.Sottile@nottingham.ac.uk,

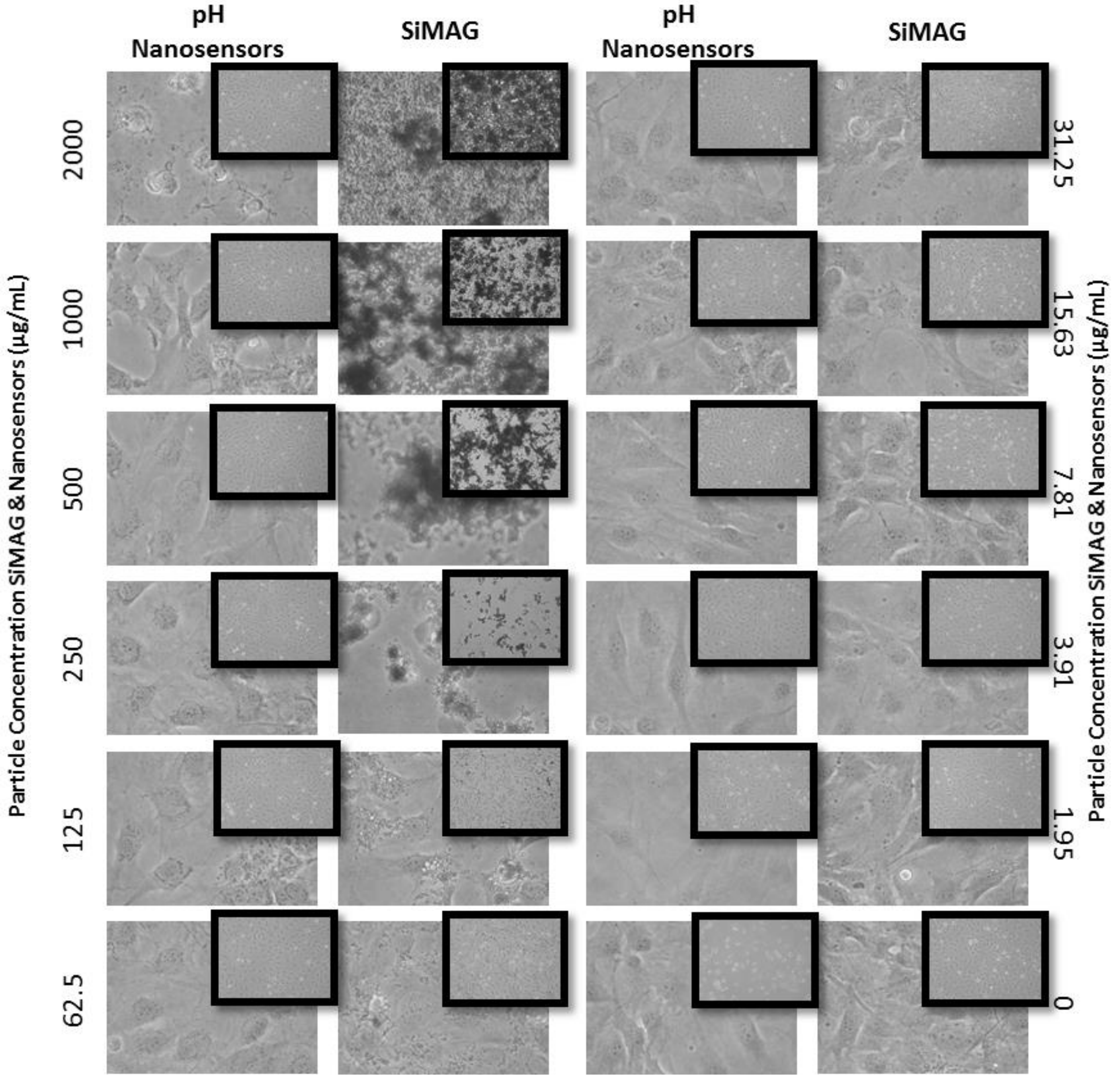

Fig. S1. hMSC morphology is preserved after loading with SiMAG particles or pH nanosensors up to 250 and 1000g/ml respectively. hMSCs were loaded with SiMAG particle or pH Nanosensors at increasing concentrations for 24 hours and imaged after 48 hours. 40x magnification with insert 20x magnification.

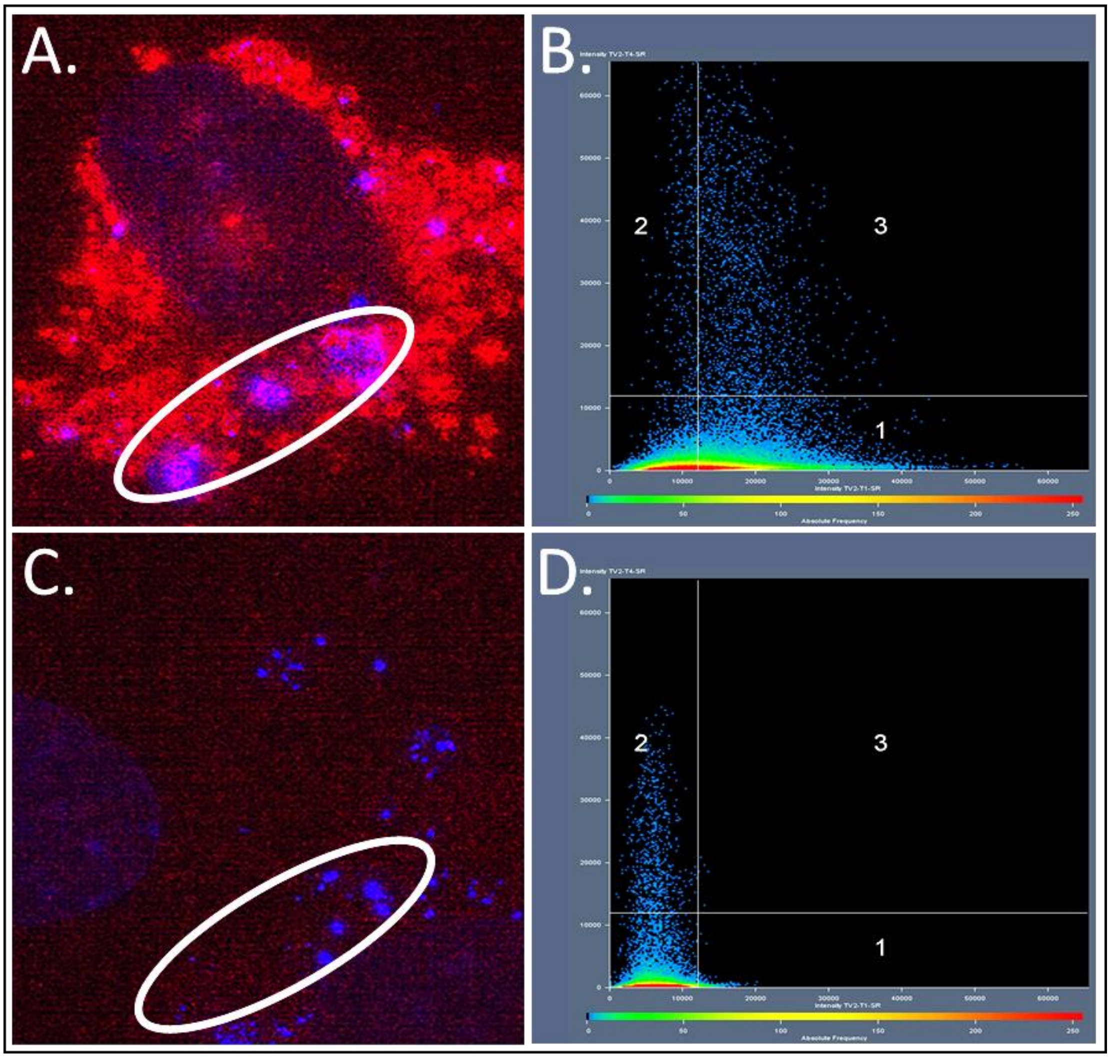

Fig. S2. SiMAG particles co-localise with lysosomal marker LAMP-1. hMSCs with and without pre-transfection with LAMP-1-RFP fusion (red), loaded with SiMAG particles (red) were imaged by super-resolution microscopy (SIM). Example images with (A) LAMP-1-RFP and (C) without LAMP-1. Region of interest highlighted by white oval. Co-localisation analysis was performed using Zen software and visualised by scatter plots, (C) region of interest with LAMP-1-RFP transfection and (D) without LAMP-1-RFP. Scatter plots show LAMP-1-RFP intensity on the x-axis and SiMAG intensity on the y-axis. Co-localisation is indicated by increased frequency in Region 3.

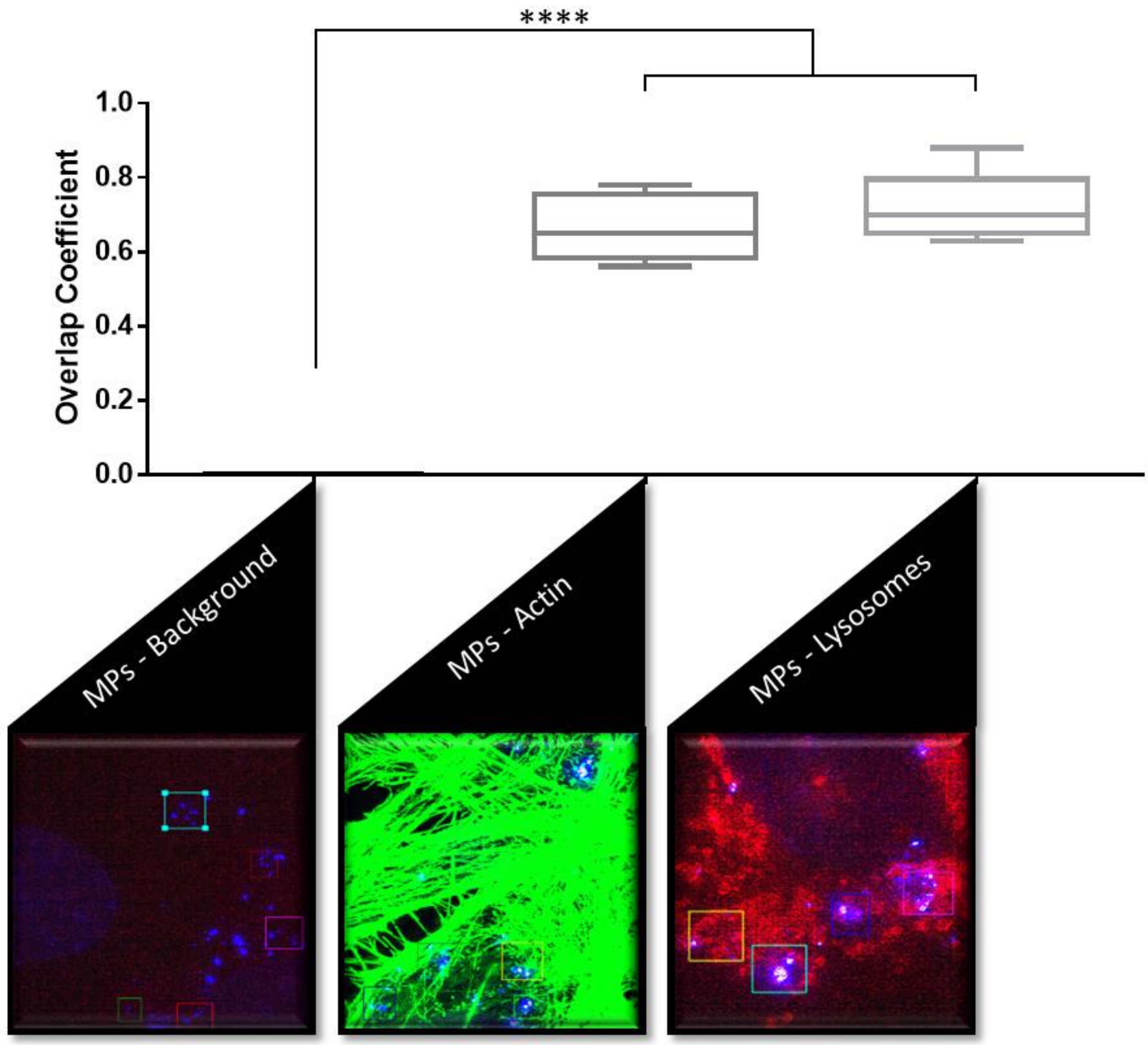

Figure S3. SiMAG particles and LAMP-1 co-localisation quantification. hMSCs pre-transfected with LAMP-1-RFP fusion and loaded with SiMAG particles were stained with phalloidin and Hoechst 33342 to identify actin cytoskeleton and nucleus respectively. Signal overlap was compared between SiMAG particle (blue) and red background, actin cytoskeleton (green) and lysosomes identified by LAMP-1-RFP (red). Co-localisation coefficients were calculated using Zen software with the actin acting as a positive control for co-localisation.

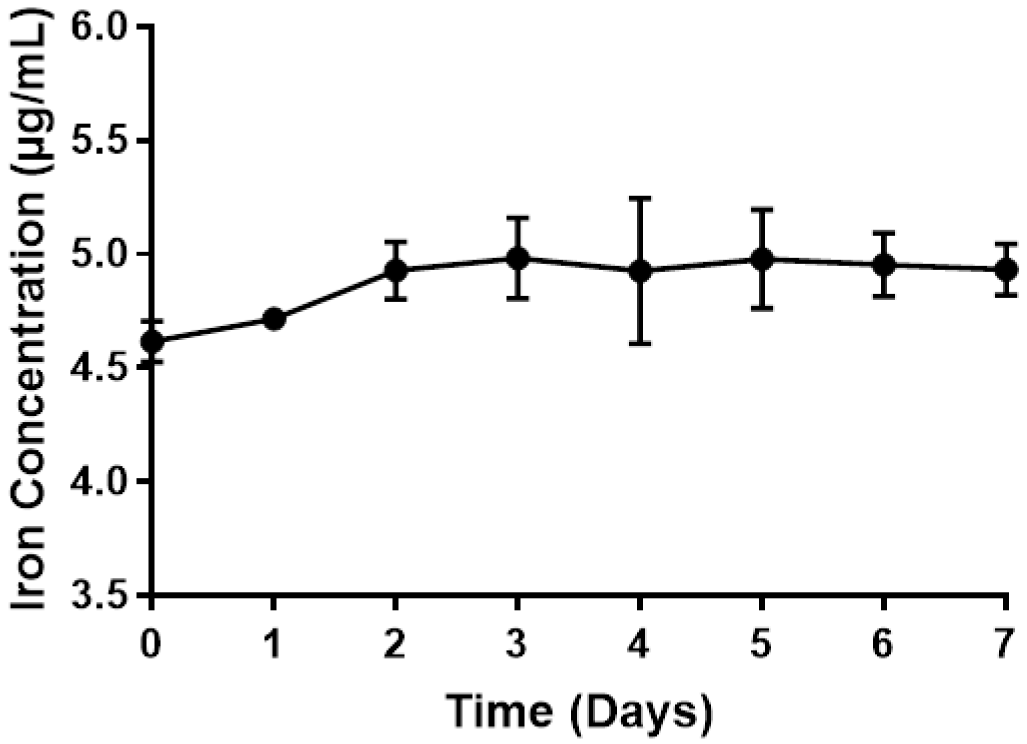

Fig. S4. Concentration of iron solubilised from SiMAG particles suspended in simulated acidic pH for 7 days determined using ICP-MS (error bars are standard error mean, where n=3).

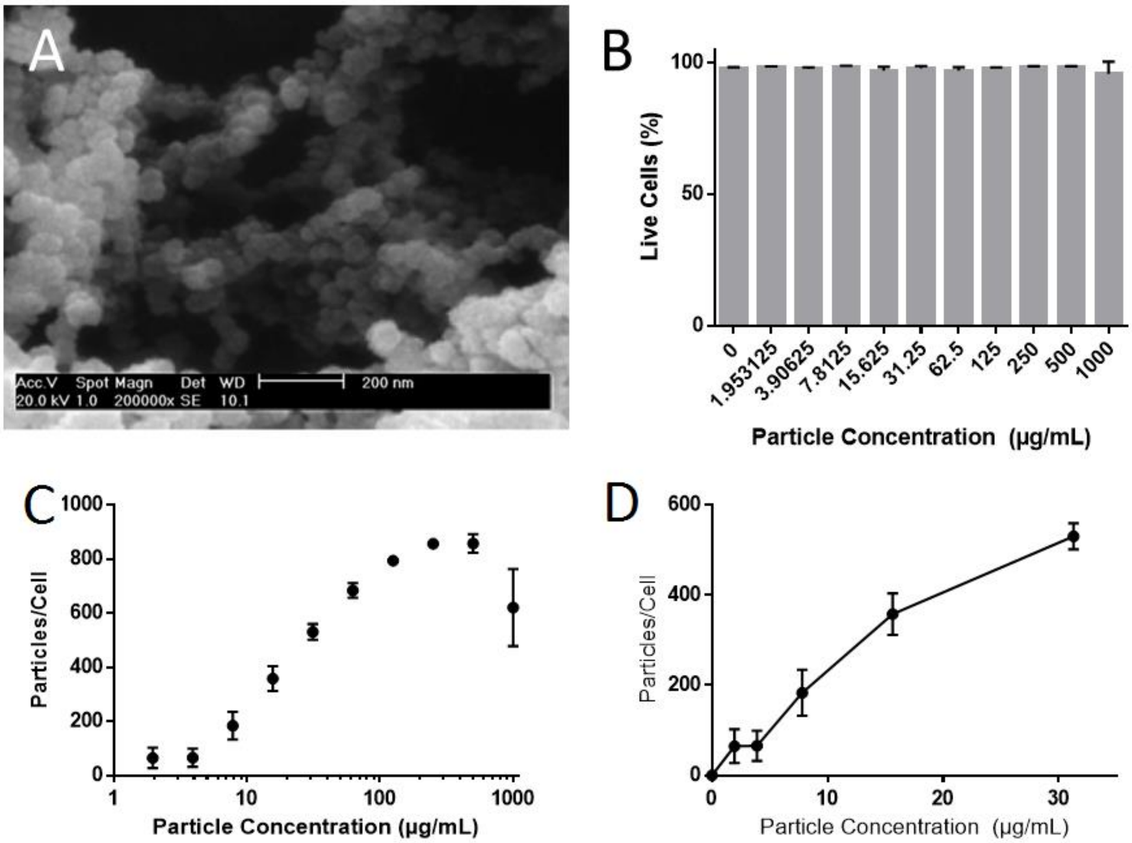

Fig. S5. pH nanosensor characterisation, cellular viability and loading of hMSCs (A) SEM imaging of pH nanosensors. (B) Cell viability of hMSCs loaded with pH nanosensors was determined by membrane permeability assay using 7-AAD and flow cytometric analysis. Utilising the fluorescence signal of the pH nanosensors the number of nanosensors within each cell was determined with flow cytometry. Flow cytometry was utilised to calculate the number of particles within each cell using the fluorescence of the dye, for (C) full range of particle loading and (D) standard working concentrations, where error bars = SD (n=3).

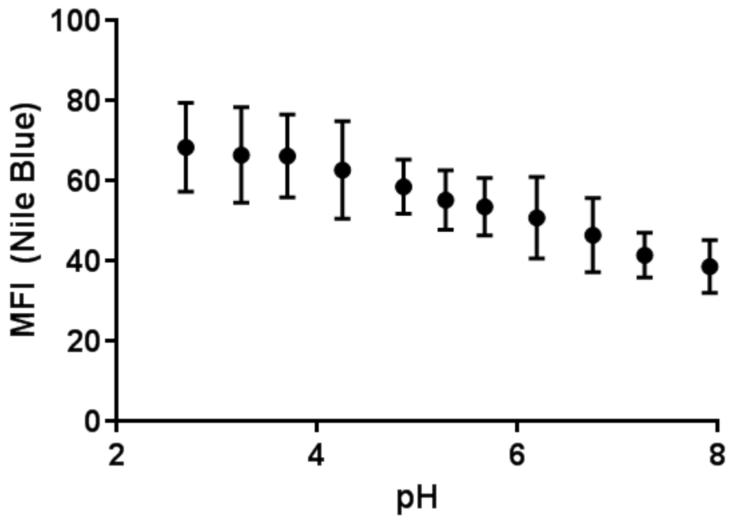

Fig. S6. SiMAG fluorescence response to changes in pH, where error bars = standard deviation (n=3).
